# Changes in the Gonial Angle Following Bilateral Sagittal Split Osteotomy and Vertical Ramus Osteotomy for Mandibular Excess

**Published:** 2010-02-23

**Authors:** Javad Yazdani, Kourosh Taheri Talesh, Mohammad Hosein Kalantar Motamedi, Mohammad Ali Ghavimi

**Affiliations:** ^a^Department of Oral and Maxillofacial Surgery, Faculty of Dentistry, Tabriz University of Medical Sciences, Tabriz, Iran; ^b^Trauma Research Center, Baqiyatallah University of Medical Sciences, Tehran, Iran; ^c^OMS Department, Azad University of Medical Sciences, Dental College, Tehran, Iran

## Abstract

**Aim:** The gonial angle plays an important role in ensuring a harmonious facial profile. Changes in this angle after surgery may be an esthetic concern for both the patient and the surgeon. The aim of the present study was to evaluate gonial angle changes after mandibular setback by the bilateral sagittal split osteotomy (BSSO) and vertical ramus osteotomy (VRO) techniques. **Methods:** Fifty-eight male patients with mandibular prognathism only were treated from 2004 to 2006 (deformities such as discrepancy of jaws, mandibular setback of more than 10 mm, asymmetry, and vertical discrepancy were excluded). Patients were randomly divided into 2 groups. In the first group, mandibular setback was performed using the Obwegeser technique and wire osteosynthesis with 4 weeks' fixation (IMF), and in the second group, mandibular setback via VRO without wire osteosynthesis and 4 weeks' IMF was carried out. Lateral cephalograms were obtained for all the patients before surgery (*T*_0_) and 1 year after surgery (*T*_1_). Gonial angle and occlusal plane-SN in *T*_0_ and *T*_1_ were evaluated. **Results:** After surgery, the gonial angle had decreased in all patients. Decrease in the gonial angle in the VRO group was greater than the BSSO group. The average decrease in the gonial angle was significantly more (*P* < 0.05) in the VRO group (7°) than in the BSSO group (2°). **Conclusion:** Gonial angle decrease was observed in the present study following mandibular setback by the VRO and BSSO techniques. This decrease in the VRO group was significantly greater.

Orthognathic surgeries are performed with the intent to enhance both function and esthetics.^1^ Bilateral sagittal split osteotomy (BSSO) and vertical ramus osteotomy (VRO) are common techniques used for the correction of mandibular prognathism. Both techniques have benefits and drawbacks, as well as pros and cons. From the esthetic point of view, the mandibular or gonial angle plays an important role. It is an important factor in ensuring a harmonious facial profile.^2^ Both BSSO and VRO reduce the gonial angle following setback.^3^^-^5 The aim of this study was to evaluate changes in gonial angles following mandibular setback by the BSSO and VRO techniques.

## MATERIALS AND METHODS

Sixty-four patients with mandibular prognathism only and mandibular excess less than 10 mm without open bite or deviation were treated. In one group, mandibular setback was performed by the Obwegeser technique and wire osteosynthesis of the upper border with 4 weeks' intermaxillary fixation (IMF), and in the other group, mandibular setback by the VRO technique without wire osteosynthesis and 4 weeks' IMF was carried out. Lateral cephalograms were assessed in all patients before the surgery (*T*_0_) and 1 year after surgery (*T*_1_). Gonial angle and occlusal plane-SN plane at *T*_0_ and *T*_1_ were evaluated.

## RESULTS

**Figure 1:**
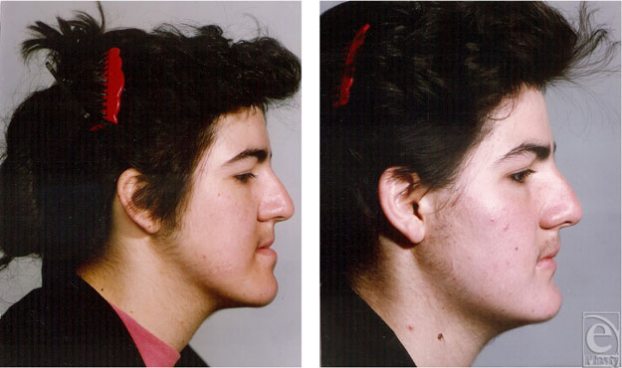
A typical patient with mandibular prognathism: (left) preoperative profile; (right) postoperative profile. Note that although the mandible has been setback following bilateral sagittal split osteotomy, the gonial angle is still high and steep.

**Figure 2:**
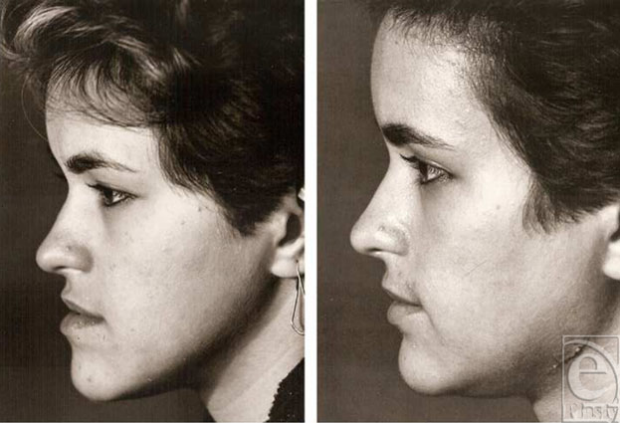
A typical patient with mandibular prognathism: (left) preoperative profile; (right) postoperative profile. Note that the mandible has been setback following vertical ramus osteotomy, and the steep gonial angle has been decreased.

The study group consisted of 58 male patients between 18 and 35 years of age, with the average age of 34 years. There were 29 patients in the first group and 29 patients in the second group. The average gonial angle in the first group before surgery was 136 ± 5° and the average gonial angle in the second group before the surgery was 134 ± 5°. After the surgery, the gonial angle had decreased in all patients. The average gonial angle in the first group was 134 ± 5° at *T*_1_ (Fig 1) and in the second group was 127 ± 5 at *T*_1_ (Fig 2), which was significantly less than in the first group. The average decrease in gonial angle in the first group was 2° and in the second group was 7°. The change in occlusal plane angle at *T*_0_ and *T*_1_ was not significant in either group.

## DISCUSSION

Obwegeser developed the BSSO technique and stated that the gonial angle decreased during mandibular setback. Previous studies conducted to evaluate gonial angle changes and its relapse rate concluded that the use of the Obwegeser setback technique caused a decrease in the gonial angle. Singer and Bays^4^ and Gomes and Wisteh^5^ in different surveys concluded that the gonial angle increases with mandibular advancement. Because the gonial angle plays an important role in ensuring a harmonious facial profile, changes in this angle after surgery may be an esthetic concern for both the patient and the surgeon. Gu et al^6^ performed sagittal split ramus osteotomies on 62 patients and showed a 2.6° reduction in the gonial angle, which was similar to that achieved in the current investigation. In a recent study,^7^ it was shown that considerable remodeling of the bilateral gonial angle also occurs after surgery. Yoshioka et al^8^ reported a significant correlation between the amount of setback and the amount of lateral gonial deviation in the VRO technique. However, stability after VRO was equal to that after SSRO.^8^ Vertical ramus osteotomy (intraoral) offers some advantages, such as lower chance of nerve damage, over SSRO for the treatment of prognathic patients.^8^ Apart from other parameters, and with regard to profile from an esthetic point of view, the mandibular or gonial angle may be more harmonious following VRO mandibular setback.^9^ We sought to assess the impact of these 2 techniques with regard to postoperative profile. In our study, although the change in occlusal plane-SN angle was not significant, gonial angle decrease was observed following mandibular setback by both the VRO and BSSO techniques. Although the lips, sella-nasion A, and sella-nasion B angles were corrected postoperatively in both groups, the decrease in gonial angles was significantly greater in the VRO group. Although VRO creates a better profile and significantly less possibility of alveolar nerve damage intraoperatively, it is used less frequently today.^10^^-^12 The ability to rigidly fixate the segments and a less need for maxillomandibular fixation postoperatively are invaluable features of BSSO for mandibular prognathism despite inherent drawbacks.

## CONCLUSION

Our study showed that in patients requiring greater reduction in the gonial angle, the VRO technique may be considered for mandibular setback.
